# A Pair of Partially Overlapping *Arabidopsis* Genes with Antagonistic Circadian Expression

**DOI:** 10.1155/2012/349527

**Published:** 2012-04-03

**Authors:** Andrea Kunova, Elena Zubko, Peter Meyer

**Affiliations:** Centre for Plant Sciences, University of Leeds, Leeds LS2 9JT, UK

## Abstract

A large number of plant genes are aligned with partially overlapping genes in antisense orientation. Transcription of both genes would therefore favour the formation of double-stranded RNA, providing a substrate for the RNAi machinery, and enhanced antisense transcription should therefore reduce sense transcript levels. We have identified a gene pair that resembles a model for antisense-based gene regulation as a T-DNA insertion into the antisense gene causes a reduction in antisense transcript levels and an increase in sense transcript levels. The same effect was, however, also observed when the two genes were inserted as transgenes into different chromosomal locations, independent of the sense and antisense gene being expressed individually or jointly. Our results therefore indicate that antagonistic changes in sense/antisense transcript levels do not necessarily reflect antisense-mediated regulation. More likely, the partial overlap of the two genes may have favoured the evolution of antagonistic expression patterns preventing RNAi effects.

## 1. Introduction

The expression of genetic information involves a sequence of molecular processes, including transcript synthesis, processing, turnover, transport and translation. Eukaryotes have developed regulatory systems at each of these levels, which can all contribute to the expression efficiency and profile of a gene. Key components in this context are natural antisense transcripts (NATs), for which a regulatory role has been demonstrated at most levels [1]. Work on yeast and animals has identified model genes that illustrate the regulatory influence of antisense transcription on sense transcript synthesis, processing, or stability. This has led to the proposal that NATs have evolved to comprise a second tier of gene expression in eukaryotes [2].

Most of our current knowledge about the mechanistic aspects of antisense-mediated gene regulation derives from work in the fungal or animal field. In plant research, antisense-mediated gene regulation systems are poorly characterised, which is contrasted by an unexpectedly high number of NATs in plants [3–5]). Among 26,939 annotated *Arabidopsis* genes, 3,027 contain antisense transcripts that are jointly expressed with their sense transcript partner. For another 7,598 genes, sense and antisense transcripts are both detected in specific tissues [3]. The abundance of NATs very strongly suggests that they are part of regulatory systems. The few examples, for which natural plant antisense transcripts have been documented to modify sense transcripts, are based on RNA interference (RNAi) mechanisms that involve dsRNA degradation into small natural antisense transcript siRNAs (nat-siRNAs). The *P5CDH* gene, which encodes a stress-response regulator, and overlaps in antisense orientation with the salt-inducible *SRO5* gene. Expression of sense and antisense transcripts results in the production of nat-siRNAs that cleave the *P5CDH* transcript [6]. Another class of nat-siRNAs, which is induced by the bacterial pathogen *Pseudomonas syringae *carrying effector avrRpt2, represses PPRL, a putative negative regulator of the RPS2 resistance pathway [7]. The biogenesis of both nat-siRNAs requires RNA-dependent RNA Polymerase 6 (RDR6), which probably synthesises secondary dsRNA substrates.

To identify novel target loci for nat-siRNA regulation, we selected T-DNA insertions in antisense genes of NATs and tested how changes in antisense transcription affect sense transcript levels. We identified a locus where the T-DNA insertion did not only alter antisense transcript levels but also transcript levels of the associated sense gene. This resembles the effects of an RNA interference mechanism between sense and antisense transcripts, but, surprisingly, we find no indication for a regulatory influence of the antisense transcription on the sense transcript.

## 2. Materials and Methods

### 2.1. Insertion Lines and Plant Material

Lines SALK_048899 [8] and SM_3.32080 [9] T-DNA insertion lines were obtained from the Nottingham *Arabidopsis* Stock Centre [10] (http://arabidopsis.info/). Plants were grown in a growth chamber under short day conditions (8 hours light, 16 hours dark, temperature 22°C and the humidity 60%). Leaf tissues were collected after four weeks and stored at −80°C. Seedlings used for the expression analysis were grown on 1/2 MS medium (Duchefa Biochmie, Haarlem, The Netherlands) with 1% sucrose (Fisher Chemicals, Loughborough, UK) and grown in long day conditions (16 hours day and 8 hours night) at 22°C. After 7 days tissues were collected and immediately frozen in liquid nitrogen. Tissues were stored at −80°C.

Seedlings used for the circadian analysis were grown according to [11]. Seeds were sterilized and sown on MS medium supplemented with 3% sucrose. Plates were kept at 4°C for four days and were transferred into a growth chamber with a cycle of 12 hours light and 12 hours darkness, at a constant temperature of 22°C and 60% humidity. After 7 days seedlings were shifted to constant white light (24 hour day). Tissues were harvested after 24 hours in constant light every four hours over a 48-hour period.

### 2.2. Genotyping of Insertion Lines

Genomic DNA for genotyping was extracted from 3-4 week-old leaf tissue according to [12]. After 1 hour incubation in the extraction buffer (200 *μ*L total), samples were cleaned up with 200 *μ*L phenol : chloroform : IAA (12 : 12 : 1). Sequence data were obtained from TAIR [13]. The presence of the T-DNA insertion and the homozygosity of insertion lines was assessed by two PCR reactions using the GoTaq master mix (Promega, Madison, USA). A first PCR was performed with the forward and reverse gene-specific primers, and in a second PCR an appropriate gene specific primer (forward or reverse) was used together with the T-DNA insertion specific primer. For the SALK insertion line, the T-DNA primer 5′-AACCAGCGTGGACCGCTTCTG-3′, forward primer 5′-AGCGAACGGTGGACAGAAAC-3′, and reverse primer 5-AGAAGTTTCGAGATCATCGTC-3′ were used. For the SM line, the T-DNA primer 5′-TACGAATAAGAGCGTCCATTTTAGAGTGA-3′ forward primer 5′- ATCTACAACAATGGCTGGAG-3′, and reverse primer 5′-TGGTTCCGAGAGAACCCTTC-3′ were used. Line* rdr6-11* (At3g49500) is a substitution mutant in *Columbia* background with C- > T substitution at position 805 bp downstream of ATG. Genotyping was performed as described by [14].

### 2.3. RNA Extraction and cDNA Preparation for Expression Analysis

Total RNA for the expression analysis was extracted from seedlings or 4-week-old leaves as described by [15]. Samples used in the experiments were always collected at the same time point, as the expression of genes with circadian activity was assessed. RNA was treated with DNase (Ambion, Austin, TX, USA) and was retrotranscribed with Superscript II Reverse Transcriptase (Invitrogen, Paisley, UK) according to manufacturer's information using the oligo dT primer 5′-GGCCACGCGTCGACTAGTACTTTTTTTTTTTTTTTTT-3′. For PCR, GoTaq Master mix was used, and for qPCR analysis, a SensiMix SYBR and Fluorescein Kitwas were utilised (Quantance, London, UK). Semiquantitative PCR was performed with F and R primers located downstream of the T-DNA insertion site. *Elongation Factor 1 alpha* (At1g07940) was used for the standardization of the amount of cDNA. For qPCR analysis, qF and qR primers were used. For circadian rhythm analysis, the *CCA1* gene (At2g46830) was used as a control of experimental conditions. Sequences of all primers are listed below.

  At1g07940,
  F primer 5′-GCGTGTCATTGAGAGGTTCG-3′,
  R primer 5′-GTCAAGAGCCTCAAGGAGAG-3′
  At3g16240,
  F primer 5′-AACCCAGCCGTCACTTTTGG-3′,
  R primer 5′-TGGTTCCGAGAGAACCCTTC-3′
  At3g16250,
  F primer 5′-CCGCGAACTGATATTGAGAAG-3′,
R primer 5′-AGAAGTTTCGAGATCATCGTC
  At1g07940,
  qF primer 5′-CTCTCCTTGAGGCTCTTGACCAG-3′,
  qR primer 5′-CCAATACCACCAATCTTGTAGACATCC-3′
  At2g46830,
  qF primer 5′-AAGGCTCGATCTTCACTGGA-3′,
  qR primer 5′-TCTCCTGCTCCATCTGAACC-3′
  At3g16240, 5′-TTCTCCGGTGGATCCATGAACC-3′
  qR primer 5′-CCAACCCAGTAGACCCAGTG-3′
  At3g16240-transgenic,
  qSF primer 5′ -TCTCCGGTGGATCCCGCATACC-3′,
  qR primer 5′-CCAACCCAGTAGACCCAGTG-3′
  At3g16250,
  qF primer 5′-ACTATGGGAAGTGTACAGTTGAG-3′,
  qR primer 5′-TTATGGCTCGGACGGTTTTGG-3′
  At3g16250-transgenic,
  qASF primer 5′-CTATGGGAAGTGTACACTGCGA-3′,
  qR primer 5′-TTATGGCTCGGACGGTTTTGG-3′.

### 2.4. Cloning and Analysis of At3g16240-At3g16250 Transgene Constructs

Promoter and central regions of the At3g16240-At3g16250 locus were cloned by PCR and individual fragments were cloned into pGreen II 0179 [16]. The At3g16240-At3g16250 locus was cloned in three parts. At3g16240 promoter (from the position 5502835 bp to 5505344 bp, according to the TAIR database, http://www.arabidopsis.org/) was amplified with primers P1-F-XhoI 5′-GTTCTCGAGAAAGATGCAAAGC-3′ and P1-R-EcoRV 5′-TGTGGTGGGATATCTTGGACCCG-3′. The central region (5505345 bp to 5508454 bp) was amplified with primers OF-EcoRV 5′-AGATATCCCACCACACCACAGAAAC-3′ and OR-XmaI 5′-ACCCCGGGTAAGAGATAAAAAGAGGCACC-3′ and At3g16250 promoter (5508455 bp to 5511469 bp) was amplified with primers P2-F-XmaI 5′-TACCCGGGGTGTGTATGCGCCGGTTTAG-3′ and P2-R-SacI 5′-TTTGTAACCGAGCTCAAAGAAGTGG-3′. Individual fragments were cloned into pGreen II 0179 digested with the appropriate restriction enzymes. Primers OmodF 5′-CGCGGATCC**CGCATA**CCAGCACGTTCCTTTGGACC-3′ and OmodR 5′- CGGTGTACA**CTGCGA**TGGTTCCGGCCTAGTAGCTTC-3′ were used to amplify a fragment that was cloned into the *BamH*I and *BsrG*I digested ORF-pGreen0179 construct. At two positions of the central fragment, 6 nucleotides were changed to distinguish the endogenous gene from the transgene. Constructs containing the central region linked to the At3g16240 promoter or the At3g16250 promoter, respectively, were transformed into *Arabidopsis thaliana* Col-0 and single-copy transgenic lines of each construct were identified by Southern blot analysis. For qPCR analysis of transgenic lines, primers qSF and qASF were used, which contained the 6 bp modification at their 3′end.

## 3. Results

### 3.1. A At3g16240/At3g16250 Gene Pair with Antagonistic Changes in Transcript Levels

**Figure 1:**
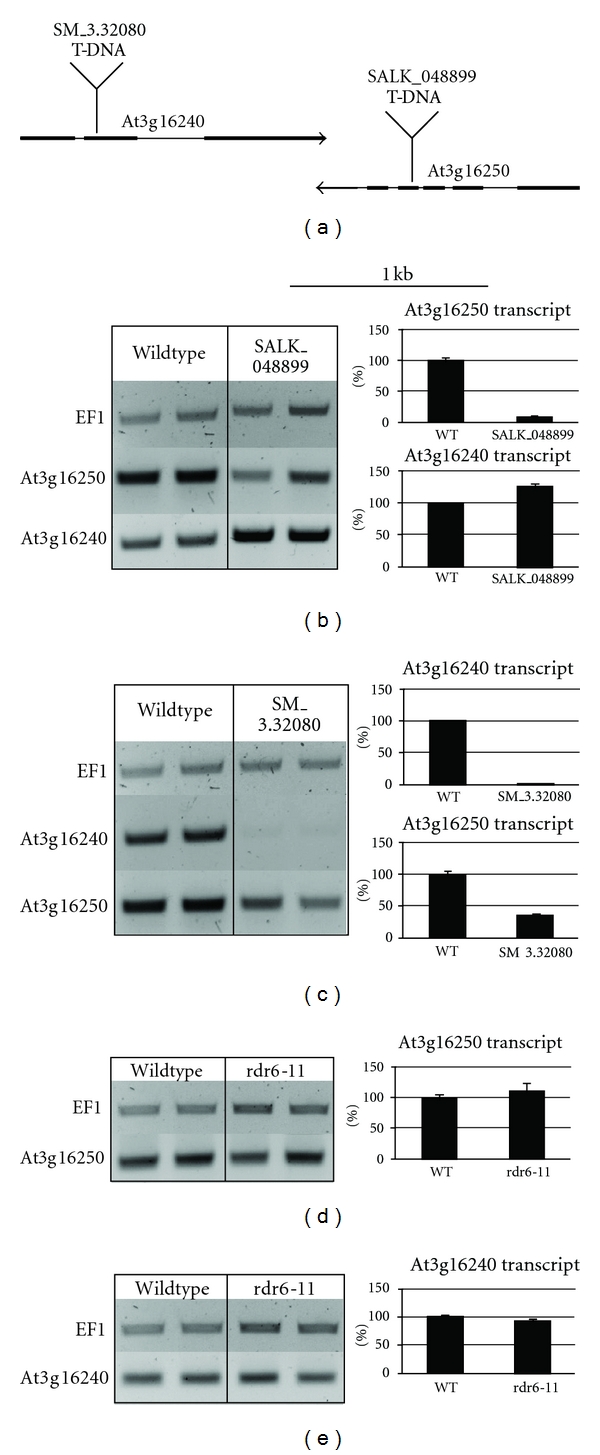
Map and transcript analysis of the locus At3g16240/At3g16250. (a) Schematic map of gene pair At3g16240/At3g16250. Thick lines indicate exons, thin lines intron regions. (b) Reduced At3g16250 transcript levels and increased At3g16240 transcript concentrations in the SALK_048899 insertion line. (c) Reduced At3g16240 and At3g16250 transcript levels in the SM3.32080 insertion line. ((d), (e)) At3g16240 and At3g16250 transcript levels are not altered in a *rdr6* mutant, compared to wildtype. The amount of cDNA of the semiquantitative PCR was calibrated to elongation factor 1 alpha (EF1a). Two replicas of semiquantitative RT-PCR data are shown (left). In the quantitative RT-PCR, wildtype levels were set to a reference level of 100% (right). The expression of both genes in wildtype and different mutants was examined at the same time point. The error bar represents standard error.

Gene At3g16240 encodes a vacuolar membrane protein, which functions as a water channel and NH_3_ transporter, and partially convergently overlaps with a gene on the opposite strand, At3g16250, which encodes a ferredoxin-related novel subunit of the chloroplast NAD(P)H dehydrogenase complex (Figure 1(a)). A T-DNA insertion into the gene At3g16250 reduces At3g16250 transcript levels and increases transcript levels of the corresponding At3g16240 gene (Figure 1(b)), while T-DNA insertion into the gene At3g16240, which almost eliminates downstream transcript levels, also reduces transcript levels of the corresponding At3g16250gene (Figure 1(c)). The joint decrease of At3g16250 transcript levels and increase in At3g16240 transcript levels are indicative of an antisense-mediated control mechanisms that involves nat-siRNA synthesis under participation of RDR6. Our analysis of the expression of both transcripts in a *rdr6 *mutant did, however, show no dependence of transcript levels on RDR6 (Figures 1(d) and 1(e)).

### 3.2. Antagonistic Circadian Activity of At3g16240 and At3g16250

**Figure 2:**
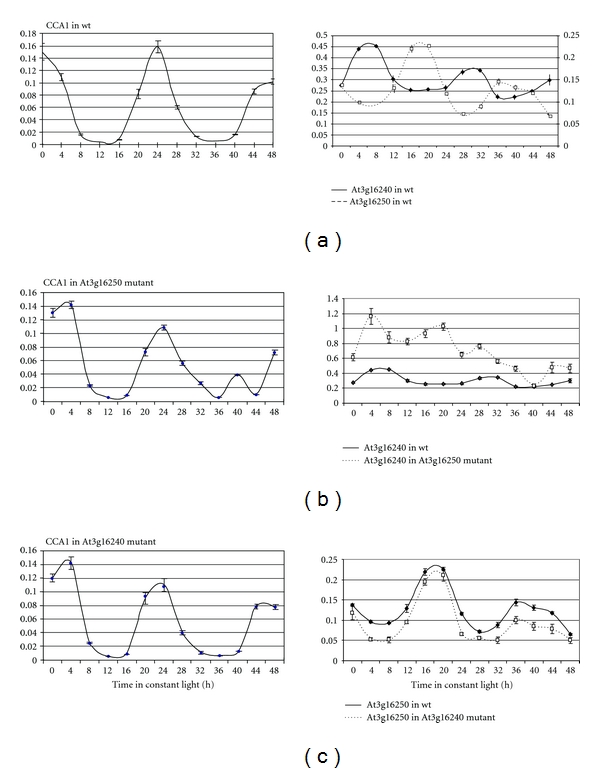
Circadian expression profile of At3g16240 and At3g16250 transcripts in wildtype and T-DNA insertion lines. *CCA1* transcript profiles were used as control of experimental conditions. The experiment was repeated two times in three replicas. The error bars represent standard error. (a) Antagonistic expression of At3g16240 and At3g16250 transcripts in wildtype. (b) Expression profile of the At3g16240 gene in wt and in the SALK_048899 insertion line. At3g16240 transcript levels are increased in the insertion line. (c) Expression profile of the At3g16250 gene in wt and in the SM3.32080 insertion line. At3g16250 transcript levels are slightly reduced in the insertion line.

The antagonistic expression profile of At3g16240 and At3g16250 is also apparent from their circadian activity. Both genes have a circadian expression pattern with a periodicity of 20–22 hours At3g16240 transcript maxima correlating with At3g16250 transcript minima, and vice versa (Figure 2(a)). This expression pattern might indicate antagonistic regulatory effects between these two transcripts. To assess if the circadian rhythm of the two genes was dependent on the expression profile of the partner gene, we compared the two transcript levels in wildtype and T-DNA insertion lines. As observed before, down-regulation of the At3g16250 transcript correlated with an increase in At3g16240 transcript levels. The circadian activity of the At3g16240 gene in the At3g16250 insertion line seems to be abolished, as no circadian cycling in transcript levels was detected after an initial peak in expression (Figure 2(b)). Reduction of At3g16240 transcript levels in the SM3.32080 mutant line correlated with a small reduction in At3g16250 transcript levels, and moreover, circadian rhythm of the At3g16250 transcript was unchanged (Figure 2(c)). While this analysis excludes an influence of the At3g16240 transcript on the circadian activity of the At3g16250 gene, the results are less conclusive with respect to an influence of the At3g16250 transcript on circadian activity of the At3g16240 gene. Either the reduction of At3g16250 transcription or the insertion of the T-DNA in the At3g16250 gene could interfere with circadian expression of the At3g16240 gene in the SALK_048899 insertion line.

**Figure 3:**
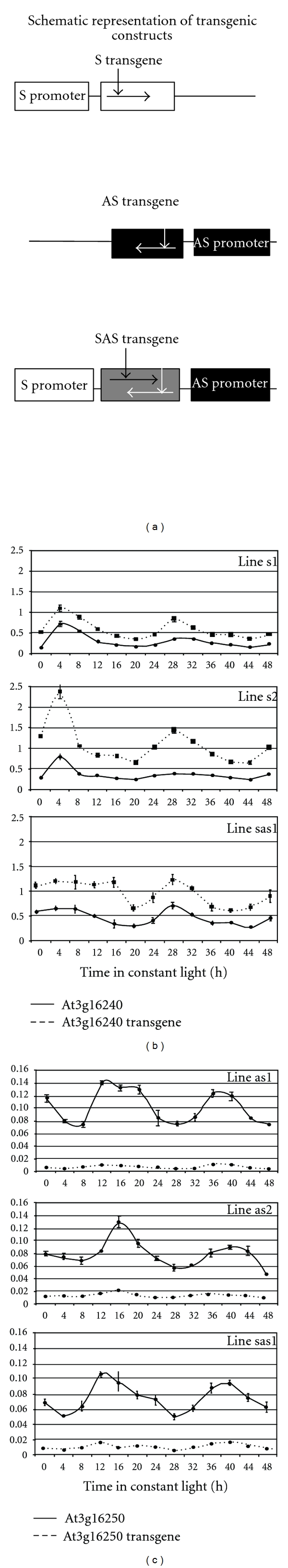
Transcript analysis of endogenous and transgenic transcripts of the At3g16240 and At3g16250 genes in transgenic lines. (a) Schematic representation of transgenic constructs used. Promoter regions and the coding region are shown. In the coding region, possible transcription of the sense or antisense transgene driven by the appropriate promoter is indicated by arrow. 6 bp substitutions in the coding region of the sense and antisense transgenes are indicated by vertical arrows. (b) In two sense transgenic lines, s1 and s2, transcript levels of the transgene are increased compared to the endogenous copy of the At3g16240 gene. The same increase occurs in a sas transgene that jointly expresses sense and antisense transgene. (c) In two antisense transgenic lines, as1 and as2, levels of the antisense transgene transcripts are decreased compared to the endogenous At3g16250 copy. The same decrease occurs in a sas transgene that jointly expresses sense and antisense transgene. The experiment was repeated twice with two replicas. The error bars represent the standard error.

To differentiate between T-DNA effects and effects from antisense transcription, we focused on a transgenic approach that examined expression of the two genes in the presence and absence of the corresponding antisense transcript. We designed a sense transgene construct (S transgene) that contained the complete At3g16240 gene without the At3g16250 gene promoter, and an antisense transgene construct (AS transgene) that contained the At3g16250 gene without the At3g16240 gene promoter. A third construct contained both genes with both promoters (SAS transgene). Six point mutations were introduced into the transgene constructs to provide primer regions for a separate analysis of transgene and endogen transcripts. The analysis of transformants with single copies of the transgenes confirmed previous results from the T-DNA analysis. The sense transgenic line expressed increased sense transgene levels compared to the endogenous At3g16240 copy, and antisense transgene levels were reduced compared to the endogenous At3g16250 copy that was linked to the At3g16240 gene (Figure 3). Both transgene constructs maintained the circadian transcript profiles, indicating that interactions between At3g16240 and At3g16250 transcripts are not required for the circadian expression profile of the two genes and that the respective promoters are able to drive the circadian expression without the need of the presence of the corresponding antisense partner. Transcript profiles of the sense and antisense transgenes were also independent of transcription in opposite orientation as transgene expression levels did not differ among transgenes expressing one or both genes. This suggests that neither the antagonistic circadian activity of the two genes, nor changes in the transcript levels observed in the insertion lines, is caused by antisense regulation.

## 4. Discussion

The coexpression of a sense transcript and its natural antisense transcript can favor the formation of dsRNA substrates, which is degraded to nat-siRNAs that can silence sense transcripts in *cis* or transcripts from homologous loci in *trans*. Degradation of the P5CDH transcript after salt induction of its NAT SRO5 requires DCL1 and DCL2, two members of the RNase III family of nucleases that specifically cleave double-stranded RNAs, and depends on the RNA-dependent RNA polymerase RDR6, SGS3, and NRPD1A. In a two-stage process, a 24-nt siRNA is formed by a biogenesis pathway dependent on DCL2, RDR6, SGS3, and NRPD1A, which establishes a phase for the generation of 21-nt siRNAs by DCL1 and further cleavage of P5CDH transcripts [6]. Generation of a ~22 nt endogenous siRNA, nat-siRNA ATGB2, induced by *Pseudomonas syringae*, also requires RDR6, NRPD1A, and SGS3, but only one DICER enzyme, DCL1 [7]. A third example is the *Sho* gene from *Petunia hybrida,* which encodes an enzyme responsible for the synthesis of plant cytokinins. The distribution of two pools of *Sho*-specific small RNAs suggests that a partially overlapping antisense transcript can be activated in a tissue-specific response to adjust local cytokinin synthesis via degradation of* Sho* dsRNA [17].

A common feature of the two nat-siRNA producing loci in *Arabidopsis* is their sensitivity to changes in sense and antisense transcript levels, a feature that we also observe for the gene pair At3g16240/At3g16250. Reduction of At3g16250 transcript levels in a T-DNA mutant SALK_048899 correlated with an increase in At3g16240 transcript levels. The expression of the two genes was not altered in a *rdr6* mutant, which argued against an RDR6-mediated nat-siRNA pathway but it did not exclude an RDR6-independent RNA degradation mechanisms. An alternative mechanism for antagonistic effects between antisense and sense transcription would be transcriptional interference, which is based on the suppressive influence of a transcriptional process in *cis *on a second transcriptional process [18] as it was demonstrated for the *GAL10* and *GAL7* genes in *Saccharomyces cerevisiae*. When both genes were arranged as convergently overlapping pairs, transcription initiation was unaffected but transcription elongation was severely inhibited. The effect was only observed in *cis*, and was proposed to reflect a collision of the two polymerase complexes [19].

The possibility of antisense-mediated gene regulation was further supported by the observation of an antagonistic circadian activity of At3g16240 and At3g16250 gene. This offered an attractive model for antisense-mediated regulation as NATs complementary to clock gene transcripts have been reported in mammals, insects, and fungi [20]. A direct effect of antisense transcription on the circadian activity of the sense transcript has been demonstrated for the *frq* locus in* Neurospora crassa. *Antisense transcript levels of the* frq *transcript cycle in antiphase sense *frq* transcripts in the dark and are inducible by light. Inhibition of antisense transcription in mutant strains alters the time and resetting of the clock [21]. Our analysis of transgene construct does, however, not support a regulatory role of antisense transcription in cyclic expression of At3g16240 or At3g16250, as their circadian expression profile is not altered in plants that express At3g16240 or At3g16250 transgenes with or without antisense transcription.

Our results therefore suggest that joint antagonistic changes in At3g16240 and At3g16250 transcript levels must not necessarily be based on dsRNA degradation or transcriptional interference. The joint sense transcript increase and antisense transcript decrease is more likely the consequence of removing the two genes from its chromosomal environment. We speculate that transcriptional competence of the two convergently overlapping genes is influenced by the local chromatin environment, which induces a more active conformation on the At3g16250 gene then on the At3g16240 gene. This influence may be mediated by chromatin domain or isochore structure [22], interaction with the nuclear scaffold [23], epigenetic marks [24], chromatin torsion [25], or other effects that influence transcriptional competence. Both T-DNA integration and translocation could disturb this local chromosomal impact on gene expression and cause the observed antagonistic changes that mimic RNAi effects.

Our results do not challenge the phenomenon of the existence and significance of antisense-mediated gene regulation but they highlight that antagonistic expression patterns must not necessarily be the consequence of antisense transcription. Partially overlapping sense-antisense gene pairs may have coevolved with mechanisms that prevent interference between their transcripts. One example is the evolution of alternative polyadenylation regions that shorten sense transcript length and prevent transcript overlap with antisense transcripts [26]. The presence of a partly overlapping gene in antisense orientation may favour the evolution of antagonistic expression patterns, which would avoid the generation of large amounts of double-stranded RNA as substrates for RNA degradation mechanisms.
